# The difficulty of continuing sports activities after open-wedge high tibial osteotomy in patient with medial knee osteoarthritis: a retrospective case series at 2-year-minimum follow-up

**DOI:** 10.1186/s40634-021-00385-4

**Published:** 2021-08-25

**Authors:** Shugo Maeda, Daisuke Chiba, Eiji Sasaki, Tetsushi Oyama, Tomoyuki Sasaki, Hironori Otsuka, Yasuyuki Ishibashi

**Affiliations:** 1 Department of Orthopaedic Surgery, Aomori Rosai Hospital, 1 Minamigaoka, Aomori 031-8551 Shirogane, Hachinohe, Japan; 2 Department of Orthopaedic Surgery, Hirosaki Memorial Hospital, Hirosaki, Japan; 3 Department of Orthopaedic Surgery, Hirosaki University Graduate School of Medicine, Hirosaki, Japan; 4 Department of Orthopaedic Surgery, Japan Community Health care Organization Akita Hospital, Noshiro, Japan

**Keywords:** Open-wedge high tibial osteotomy, Return to sport, Sports continuation rate, Knee osteoarthritis, Sports activity, Prognosis, Osteotomy around the knee

## Abstract

**Purpose:**

This study aimed to investigate the rate at which patients returned to sports after open wedge high tibial osteotomy and identify the continuity of sports activity post-operatively.

**Methods:**

Thirty-five patients (40 knees) who underwent open-wedge high tibial osteotomy (OW-HTO) in medial knee osteoarthritis were included in this study. The mean age of the patients who underwent surgery was 55.1 ± 10.7 years, and the mean follow-up period was 41.0 ± 24.7 months. Clinical results and radiographic parameters calculated in standing whole-leg radiographs preoperatively, post-operatively, and at the final follow-up were evaluated.

**Results:**

Thirty-one patients (88.6%) were able to return to preoperative sports activity; however, only 14 patients (40.0%) completely returned to preoperative sports activity levels. Of the 31 patients who returned to sports activity, 10 patients (32.3%) maintained post-operative sporting activity levels at the final follow-up. In radiographic parameters, the weight-bearing line ratio was considered loss of correction in the post-operative period leading to the final follow-up. Patients who completely returned to sports and maintained sporting activity levels at the final follow-up had significantly higher the Knee Injury and Osteoarthritis Outcome Score pain subscale values and lower visual analogue scale of knee pain at pre-surgery and final follow-up than other patients, including those who partially returned to sports.

**Conclusions:**

The proportion of patients who returned to sports after OW-HTO and were able to participate in competitions at the same activity level as before surgery was low and insufficient.

**Level of evidence:**

Retrospective case series, IV

## Introduction

The treatment of knee osteoarthritis (OA) is based on the patients’ age, body mass index (BMI), OA severity, and ligament status. Young active patients with knee OA have high functional demands and expect to return to sports and work activity post-operatively. By reducing the contact pressure on the medial tibiofemoral joint, Open-wedge high tibial osteotomy (OW-HTO) reduces pain, improves knee function, and slows knee degeneration, thereby potentially minimizing the need for knee arthroplasty [15]. Following total knee arthroplasty (TKA) and unicompartmental knee arthroplasty (UKA), low-impact sports, which have little impact on joints, are generally recommended to decrease the risk of prosthesis wear and loosening [5]. Moreover, OW-HTO with a locking compression plate accelerates rehabilitation, allows early bone union, does not cause any correction loss of radiographic findings, and induces good functional recovery [17]. Therefore, OW-HTO allows patients to participate in high-impact sports following surgery because it retains the natural joint structure [13, 14, 16]. However, details of patients’ activities after HTO, such as the sports they can play or are currently playing, as well as whether joint deformation progresses due to sports activities are largely unclear. This study aimed to investigate the rate at which patients returned to sports after OW-HTO and identify the continuity of sports activity post-operatively. We investigated the hypothesis that patients who underwent OW-HTO for medial OA have a high probability of returning to sports activities and maintaining their activity level.

## Materials & methods

### Patient selection

A total of 241 consecutive patients underwent HTO and distal femoral osteotomy using a locking plate and spacer plate for knee OA at four medical centres between April 2010 and January 2019; the patients were retrospectively assessed for eligibility for this study (retrospective case series). The study design and methods were approved by the ethics committee of each institution, and the patients provided informed consent prior to participating in this study. The inclusion criterion was a follow-up period of at least 2 years, whereas the exclusion criteria were ligament injury, a history of anterior cruciate ligament reconstruction or autologous osteochondral transplantation, and no severe patellofemoral joint OA. A total of 35 patients (14.5%; 40 knees) who underwent OW-HTO for medial knee OA and who took part in sports preoperatively were selected as subjects. One-sided OW-HTO was performed in 30 patients, whereas 5 patients underwent staged and same-day bilateral OW-HTO. Of the study subjects, 27 were males and 8 were females; the mean age of the patients who underwent surgery was 55.1 ± 10.7 years (40–76 years), and the mean BMI was 25.1 ± 3.4 kg/m^2^ (19.5–33.8 kg/m^2^). The mean period of observation was 41.0 ± 24.7 months (24.0–111.1 months); for patients who underwent OW-HTO on both knees, the follow-up period was from the date of surgery on each knee to the date of the final follow-up. The type of sports played preoperatively included running/jogging (eight patients); alpine skiing (five patients); baseball (four patients); mountain climbing and tennis (three patients each); marathon, basketball, badminton, and dancing (two patients each); karate, golf, soccer, and Nordic walking (1 patient each). In all patients, continuing sports preoperatively became difficult due to pain in the affected knee (Table 1). The Kellgren and Lawrence classifications of pre-surgical radiographs were as follows: grade 1, 5 knees; grade 2, 23 knees; and grade 3, 12 knees. Concomitant surgeries included partial meniscectomy in 9 knees, meniscal repair in 7 knees, and the microfracture of the femoral condyle in 5 knees. The mean opening size was 8.9 ± 1.4 mm (6–12 mm).

**Table 1:** Sports activity of patients

Sports activity	Number
Running / Jogging	8
Alpine skiing	5
Baseball	4
Mountain claiming	3
Tennis	3
Marathon	2
Basketball	2
Badminton	2
Dancing	2
Karate	1
Golf	1
Soccer	1
Nordic walking	1

### Surgical methods

Preoperatively, supine whole-leg radiographs were used to plan the osteotomy size for a target axial load goal of 57.5–62.5% [4]. Intraoperatively, an arthroscope was first used to evaluate the state of the cartilage, menisci, and ligaments. Depending on the condition of the knee joint, microfracture or meniscal procedures, such as a meniscectomy or meniscal repair, were performed. Then, according to a pre-surgical plan, either biplane or transverse osteotomy of the proximal tibia was completed, and intraoperative imaging was used to confirm lower extremity alignment and adjust osteotomy size. The portion of the proximal tibia that underwent osteotomy was filled with a 60% porosity β-tricalcium phosphate block and hydroxyapatite block and was fixed using a TriS Medial HTO plate (Olympus Terumo Biomaterials, Tokyo, Japan), Position HTO plate (B. Braun Aesculap, Tuttlingen, Germany), or TomoFix (DePuy Synthes, Bettlach, Switzerland).

### Post-operative rehabilitation

Post-operatively, patients were placed on a non-weight-bearing or partial weight-bearing period of 2–4 weeks and were instructed to walk using a walker or crutches. Four weeks post-surgery, knee radiographs were obtained to verify bone healing and fixation stability; thereafter, full weight-bearing was allowed. Patients who were operated on both knees at the same day used wheelchairs during transfers and underwent standing training with support of both the upper limbs, with the less painful side as the partial load. In these patients, the start of full weight-bearing walking was set in the same manner as that in patients with one-sided surgery. After 3 months, following confirmation of the healing of the external hinge portion using X-rays, patients were allowed to begin jogging. Exercise strenuousness was increased gradually, and at 6 months post-surgery, following the confirmation of bone healing in the region that underwent osteotomy using radiography, patients were allowed to resume prior sporting activities.

### Clinical and radiographic evaluation

After evaluating the activities related to returning to sports, resuming prior practice, with the ability to participate in competitions, indicated that the patient had completely returned to sports; participation in practice but with decreased frequency or quality indicated that the patient had partially returned to playing sports; and inability to practice or quitting sports indicated that the patient did not return to sports. Information regarding activities related to returning to sports was derived from telephone interviews, mail-back surveys, or clinical records (percentage of each method, 2.9%, 31.4%, and 65.7%, respectively). The types of sports pre- and post-operatively were evaluated using the Tegner activity scale. For clinical evaluation, the Japanese Orthopaedic Association (JOA) score [1, 19] was used to objectively assess the treatment outcome; the level of knee pain was assessed using a visual analogue scale (VAS) preoperatively and at the final follow-up, and the values were compared. The Knee Injury and Osteoarthritis Outcome Score (KOOS) [9] was evaluated for patient-based outcome scores at the final follow-up only. The JOA and VAS scored of each knee were evaluated, and the KOOS score was obtained to assess the condition of both knees of patients operated on both knees.

**Fig. 1:**
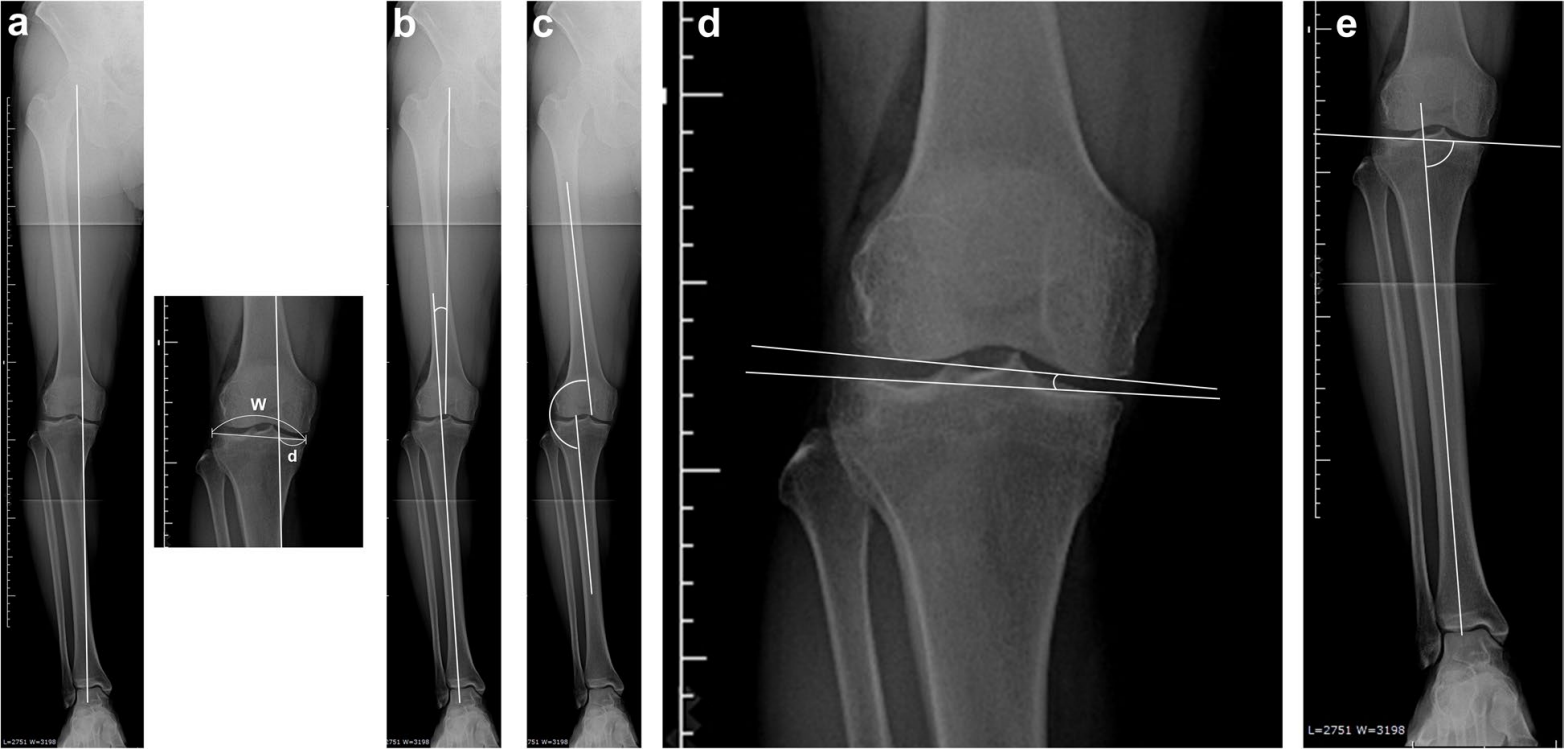
Radiographic evaluation (bilateral standing whole-leg radiographs), **a** The weight-bearing line ratio (WBL ratio). The horizontal distance from the medial edge of the tibial plateau (d), divided by the width of the tibial plateau (W), as d/W × 100%. The WBL ratio of the medial edge was regarded as 0% and the lateral edge as 100%, **b** Hip-knee-ankle angle (HKA), the angle formed by the mechanical axis of femur and tibia. Negative values are assigned to knees with varus alignment, **c** Femoro-tibial angle (FTA), **d** Joint line convergence angle, **e** Medial proximal tibial angle

**Fig. 2:**
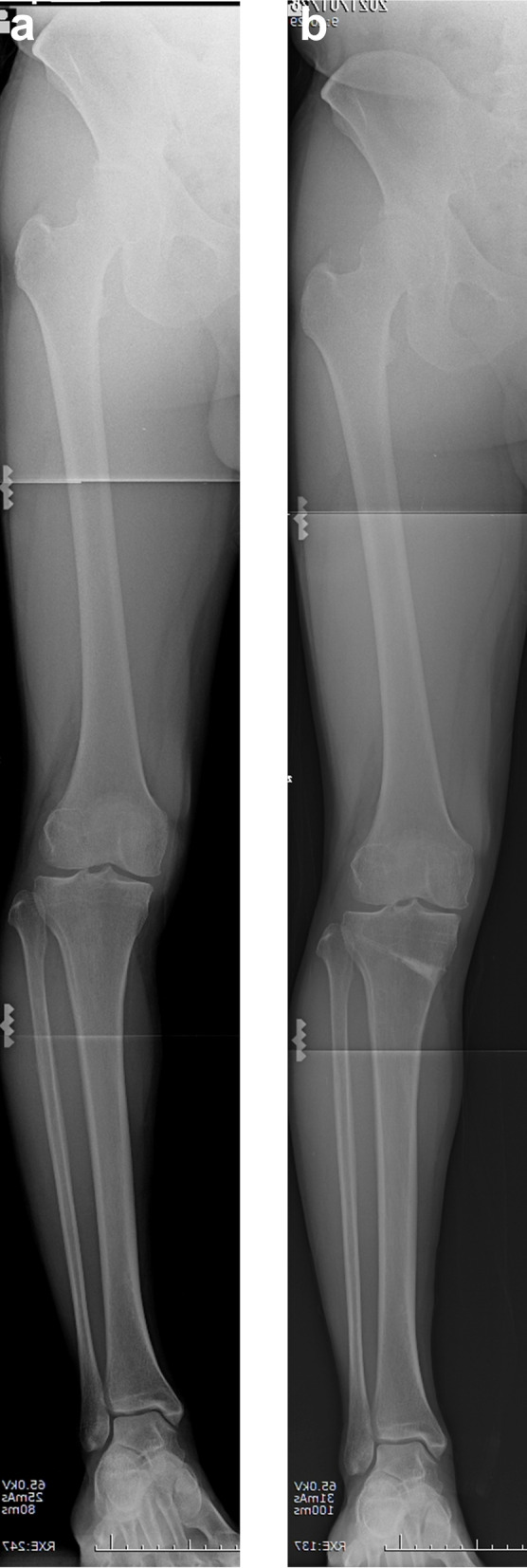
Pre- and post-operative hole bilateral standing whole-leg radiographs. **a** Preoperative radiograph, **b** Post-operative (final follow-up) radiograph

For radiographic evaluation, bilateral standing whole-leg radiographs were used to calculate the weight-bearing line (WBL) ratio, hip-knee-ankle angle (HKA), femoro-tibial angle (FTA), joint line convergence angle (JLCA), and medial proximal tibial angle (MPTA); the preoperative values, post-operative values at 1 month, and the values of these factors at the final follow-up were compared [12, 15, 17] (Fig. 1a-e and Fig. 2a-b). The WBL ratio was defined as the ratio of the distance between the medial edge of the tibial plateau and the intersection of the mechanical axis and the length of the tibial plateau. The WBL ratio of the medial edge was regarded as 0% and the lateral edge as 100%. The HKA was the angle formed by the mechanical axis of the femur and tibia. Negative values were assigned to knees with varus alignment. X-ray image evaluation and clinical evaluation of the final follow-up were performed on the same day. Four orthopaedic surgeons (S.M., D.C., E.S., and T.O.) calculated the radiographic parameters in each institution. The radiographic parameters of each patient were measured four times by each observer, and the observers were blinded to the other observers’ data.

### Statistical analysis

To achieve an 80% statistical power with an α level of 0.05 in demonstrating a medium effect size (ρ = 0.5), a power analysis using a free statistical power analysis software (G*Power, version 3.1.9.2; Frans Faul, Universität Kiel) revealed that a minimum of 64 subjects was required. The Tegner activity scale and JOA scores of all cases preoperatively and at the final follow-up were compared using the Wilcoxon signed-rank test. The WBL ratio, HKA, FTA, JLCA, and MPTA of radiographic parameters pre-surgery, post-surgery, and at the final follow-up were analysed using the Friedman test (with post-test Bonferroni correction). To assess the interrater reliability of the radiographic measurements, the interclass correlation coefficients for the interobserver agreements was calculated. Furthermore, the 35 patients included in this study were divided into two groups based on the activity level of sports post-operatively. The first group consisted of patients who completely returned to sports and maintained the activity level by the final follow-up (Group C), and the other group comprised patients who partially returned and those who have returned to sports once but have continued to lower their activity level (Group P). Baseline demographic data, the three clinical parameters (JOA score, VAS, and KOOS) at the final follow-up, and radiographic parameters were analysed using the Mann–Whitney U test and Fisher’s exact test and were compared between the two groups. The significance level was set at 5%. All statistical analyses were performed with EZR (Saitama Medical Centre, Jichi Medical University, http://www.jichi.ac.jp/saitama-sct/SaitamaHP.files/statmedEN.html;Kanda, 2012), which is a graphical user interface for R (The R Foundation for Statistical Computing, Vienna, Austria, version 2.13.0). Specifically, it is a modified version of R commander (version 1.6–3) that was designed to add statistical functions frequently used in biostatistics [7].

## Results

### Return to sports activity

Of the 35 patients who participated in sports preoperatively, 31 patients (88.6%) were able to return to previous sporting activities. Fourteen patients (40.0%) completely returned to sports, and 17 patients (48.6%) partially returned. However, four patients (11.4%) did not return to sports (two alpine skiers, one tennis player, and one basketball player). The mean period to return to sports was 9.5 ± 4.7 months (6–24 months). Of the 31 patients who returned to sports, 10 patients (32.3%) maintained post-operative sporting activity levels at the final follow-up (Group C). However, 21 patients (67.7%) continued to participate in sports at a reduced level post-operatively. These patients were defined as the partial return to sports group (Group P).

### Clinical evaluation

The average Tegner activity scale score at the final follow-up was significantly lower than the preoperative activity scale score (5.4 ± 1.6 vs 5.1 ± 1.9, *p* = 0.033). The JOA score at the final follow-up significantly improved compared to the preoperative JOA score (80.0 ± 9.2 vs. 95.3 ± 6.5, *p* < 0.001). Unfortunately, one patient underwent conversion to TKA after approximately 7 years due to affected knee pain.

### Radiographic evaluation

The inter-rater reliability of radiographic measurement was 0.83 (range, 0.79–0.86). In radiographic parameters, the WBL ratio was corrected from a pre-surgical value of 25.5 ± 10.8% to a post-surgical value of 57.7 ± 6.7% (*p* < 0.001). This was considered a loss of correction in the post-operative period leading to the final follow-up (55.6 ± 7.0%, *p* < 0.001). HKA was significantly corrected from -4.4 ± 2.7 degrees pre-surgery to 2.8 ± 1.8 degrees post-surgery (*p* < 0.001), and this correction was maintained at the final follow-up (1.3 ± 0.9 degrees, *p* = 0.700). FTA was significantly corrected from 179.7 ± 3.0 degrees pre-surgery to 171.8 ± 2.1 degrees post-surgery (*p* < 0.001), and this correction was maintained at the final follow-up (172.2 ± 2.1 degrees, *p* = 0.620). JLCA was significantly corrected from 2.5 ± 1.5 degrees pre-surgery to 1.4 ± 1.0 degrees post-surgery (*p* < 0.001), and this correction was maintained at the final follow-up (1.3 ± 0.9 degrees, *p* = 1.0). MPTA was significantly corrected from 85.1 ± 2.0 degrees pre-surgery to 90.4 ± 1.5 degrees post-surgery (*p* < 0.001), and this correction was maintained at the final follow-up (90.1 ± 1.9 degrees, *p* = 0.99).

### Comparison between group C and group P

The patient demographic data of the two groups are shown in Table 2. In clinical evaluations, the KOOS pain subscale value in group C was significantly higher than that in group P (88.7 ± 10.8 vs. 79.5 ± 15.1, *p* = 0.044). Furthermore, the VAS scores at pre-surgery and final follow-up in group P were significantly higher than those in group C (79.8 ± 22.6 vs. 65.4 ± 18.2, *p* = 0.0301, and 22.6 ± 19.8 vs. 8.5 ± 8.6, *p* = 0.048, respectively) (Table 3). No significant difference was noted in radiographic parameters between the two groups (Table 4).

**Table 2:** Demographic data of the two groups

	Group C	Group P	*P* value
Number of patients	10	21	
Number of knees	11	24	
Sex male/female	8/2	20/1	0.237
Age	51.5 ± 8.5	55.3 ± 10.9	0.319
BMI	26.0 ± 4.4	24.9 ± 3.0	0.445
Follow-up months	33.9 ± 18.2	43.3 ± 25.0	0.109
Sports			
Running, jogging	1	7	
Alpine skiing		3	
Baseball	2	2	
Mountain climbing		3	
Tennis	1	1	
Dancing	1	1	
Basketball	1		
Badminton	2		
Marathon		2	
Karate	1		
Golf		1	
Soccer		1	
Nordic walking	1		
KL-grade			0.241
Grade 1	2	3	
Grade 2	8	12	
Grade 3	1	9	
Additional Surgery			0.571
Partial meniscectomy	3	6	
Meniscal repair	1	6	
Microfracture	2	2	

Group C: Complete return to sports and maintain sports activity levels post-surgery by the time of the final follow-up

Group P: Partially return to sports and continue to sports activity at a reduced level from post-surgery

*BMI* Body mass index, *KL* Kellgren and Lawrence

**Table 3:** Clinical evaluation before and after surgery

	Group C (n = 10)	Group P (n = 24)	*P* value
JOA score			
Preoperatively	80.9 ± 6.0	80.0 ± 8.8	0.729
Final follow-up	98.6 ± 2.2	93.8 ± 7.5	0.051
KOOS			
Final follow-up			
Pain	88.7 ± 10.8	79.5 ± 15.1	0.044
Symptom	84.7 ± 9.0	82.7 ± 12.9	0.901
ADL	89.8 ± 11.7	90.9 ± 7.8	0.802
Sport/Rec	73.6 ± 27.7	67.7 ± 22.6	0.277
QOL	68.1 ± 27.8	60.3 ± 25.0	0.344
Knee pain (VAS)			
Pre-surgery	65.4 ± 18.2	79.8 ± 22.6	0.0301
Final follow-up	8.5 ± 8.6	22.6 ± 19.8	0.048

Group C: Complete return to sports and maintain sports activity levels post-surgery by the time of the final follow-up

Group P: Partially return to sports and continue to sports activity at a reduced level from post-surgery

Values are presented as mean ± SD

*JOA* Japanese Orthopaedic Association, *KOOS* Knee injury and Osteoarthritis Outcome Score, *ADL* Activities of Daily Living, *Sport/Rec* Sports and Recreation, *QOL* Quality of Life, *VAS* Visual analogue scale

**Table 4:** Radiographic evaluation before and after surgery

	Group C (n = 10)	Group P (n = 25)	*P* value
WBL ratio			
Pre-surgery	29.7 ± 8.5	24.0 ± 11.1	0.145
Post-surgery	57.2 ± 8.3	58.6 ± 5.9	0.510
Final follow-up	54.5 ± 8.8	56.7 ± 5.5	0.423
HKA			
Pre-surgery	-4.2 ± 2.4	-4.8 ± 2.7	0.111
Post-surgery	2.7 ± 1.9	3.0 ± 1.7	0.899
Final follow-up	2.1 ± 1.8	2.9 ± 1.4	0.687
FTA			
Pre-surgery	179.4 ± 2.3	180.1 ± 3.1	0.185
Post-surgery	172.3 ± 2.2	171.6 ± 2.0	0.786
Final follow-up	172.7 ± 2.1	171.9 ± 1.9	0.614
JLCA			
Pre-surgery	2.1 ± 0.9	2.8 ± 1.7	0.476
Post-surgery	1.2 ± 0.7	1.6 ± 1.2	0.363
Final follow-up	1.5 ± 0.9	1.3 ± 0.9	0.721
MPTA			
Pre-surgery	84.6 ± 1.4	85.3 ± 2.0	0.282
Post-surgery	90.3 ± 1.6	90.8 ± 1.3	0.474
Final follow-up	90.3 ± 1.3	90.0 ± 2.0	0.868

Group C: Complete return to sports and maintain sports activity levels post-surgery by the time of final follow-up

Group P: Partially return to sports and continue to sports activity at a reduced level from post-surgery

Values are presented as mean ± SD

*WBL ratio* Weight-bearing line ratio, *HKA* Hip-knee-ankle angle, *FTA* Femoro-tibial angle, *JLCA* Joint line convergence angle, *MPTA* Medial proximal tibial angle

## Discussion

The most important findings of the present study were that 31 patients (88.6%) were able to return to preoperative sports activity, and only 14 patients (40.0%) completely returned to the preoperative sports activity level. Of these 31 patients, 10 patients (32.3%) maintained post-surgery sporting activity levels at the final follow-up. Based on radiographic evaluation, lower extremity alignment (WBL ratio) was considered loss of correction in the post-operative period leading to the final follow-up. Patients who completely returned to sports and maintained sporting activity levels at the final follow-up had significantly higher KOOS pain subscale values and lower VAS scores at pre-surgery and final follow-up than other patients, including those who partially returned to sports.

Ekhtiari et al. have reported that the rate at which patients returned to sports following OW-HTO was relatively high (85.2–90.8%), whereas at professional sporting levels, this number has been reported to be lower (54%) [3]. Hoorntje et al. have reported that among 294 patients who underwent HTO, the rate at which patients returned to sporting activities was 82%, with a rate of returning to sports within 6 months post-operatively of 75% and a rate of returning to sports within 1 year post-operatively of 92%. In a logistic regression analysis, the significant prognostic factor for returning to sports activity was continued sports participation during the year before surgery [6]. In a recent study of the Japanese population, Kanto has reported that among 77 patients who underwent OW-HTO, the rate of patients who returned to sports was 89.6% (69 patients), with a mean return time of 8.7 ± 2.7 months. In these patients, 58 patients (75.3%) continued the same high-impact sports activity as they did preoperatively [8]. In our study, 31 patients (88.6%) were able to return to pre-surgical sporting activity following OW-HTO. However, only 10 patients (32.3%) maintained post-surgery sporting activity levels at the final follow-up. Compared to previous studies, the rate of patients who returned to sports was comparable. In contrast, the rate of patients who maintained the same sporting activity level was unexpectedly low. According to the pre- and post-operative patient-reported outcome measure (KOOS pain subscale and VAS of knee pain), pain may be associated with maintained sports activity. Therefore, orthopaedic surgeons should consider knee pain pre-surgery and the occurrence of knee pain post-operatively. Pre- and post-operative pain control and therapeutic intervention for knee pain (e.g., topical non-steroidal anti-inflammatory drugs (NSAIDs), oral COX-2 inhibitors and NSAIDs with proton-pump inhibitors, and intra-articular hyaluronic acid [2]) might be needed to maintain sporting activity.

HTO is a procedure in which an osteotomy is performed in the proximal tibia to correct the alignment of the lower extremity, thereby dispersing load bearing within the joint and alleviating symptoms. It is known that a target correction of 62.5% (for WBL ratio), known as Fujisawa’s point, is effective for cartilage and meniscus repair [4]. In athletes, it is crucial to avoid overcorrection, and a WBL correction ratio of 50% has been reported to be optimal [18]. In this study, with a target WBL ratio of 57.5–62.5%, OW-HTO induced good clinical outcomes with regard to returning to preoperative sports activity. However, the WBL ratio was considered a loss of correction in the post-operative period leading to the final follow-up. It is possible that deformation progresses due to sporting activities. However, this progression was found to be insignificant. Moreover, the average post-operative WBL ratio in this series was 57 degrees, which may be an insufficient correction. Compared with the positive case outcomes reported by Fujisawa et al., using a target axial load of 62.5%, insufficient correction may lead to decreased performance in the long term, whereas it has been reported that overcorrection on the tibial side may lead to excessive shear force on the tibial articular surface, leading to an increased risk of damage to the patellofemoral joint cartilage [10, 11]. As sporting activities lead to greater joint stress compared with typical daily activities, stress from sports can be expected to exert a strong effect on the joint. Therefore, unless there is a compelling reason to change the target alignment due to the sporting activity, or unless the patient is a professional athlete, it appears that a typical target alignment of 62.5% is appropriate.

This study had several limitations. First, the number of cases was small, and a post-hoc power analysis demonstrated that this investigation was underpowered to a perform statistical analysis. Therefore, the results of these analyses must be interpreted with caution. Second, it involved short-term results with variation in the length of the follow-up (minimum follow-up, 24 months; maximum follow-up, 111 months). Third, imaging evaluation was performed with plain radiographs only, rather than magnetic resonance imaging to evaluate the cartilage in detail. Fourth, the patellofemoral joint was not evaluated in the image analysis. Finally, the problem in this study was the inclusion of patients engaged several sports types and different levels of sports activity. Based on the results of this study, it is not possible to determine which sports are possible and appropriate after OW-HTO.

## Conclusion

The proportion of patients who returned to sports after OW-HTO and were able to participate in competitions at the same activity level as before surgery was low and insufficient. Patients who completely returned to sports and maintained sporting activity levels at the final follow-up had significantly higher KOOS pain subscale values and lower VAS scores at pre-surgery and final follow-up than other patients, including those who partially returned to sports. Orthopaedic surgeons should fully explain that it may be difficult to continue sports activities after OW-HTO for patients with severe pre-surgery and post-surgery pain, and it is critical to control pre- and post-surgery pain for patients who wish to continue sports activities.

## Abbreviations

OA: Osteoarthritis
OW-HTO: Open-wedge high tibial osteotomy
TKA: Total knee arthroplasty
UKA: Unicompartmental knee arthroplasty
BMI: Body mass index
JOA: Japanese Orthopaedic Association
VAS: Visual analogue scale
KOOS: Knee Injury and Osteoarthritis Outcome Score
WBL: Weight-bearing line
HKA: Hip-knee-ankle angle
FTA: Femoro-tibial angle
JLCA: Joint line convergence angle
MTPA: Medial proximal tibial angle
